# Assessing Implicit Odor Localization in Humans Using a Cross-Modal Spatial Cueing Paradigm

**DOI:** 10.1371/journal.pone.0029614

**Published:** 2011-12-27

**Authors:** Carolin Moessnang, Andreas Finkelmeyer, Alexandra Vossen, Frank Schneider, Ute Habel

**Affiliations:** 1 Department of Psychiatry, Psychotherapy and Psychosomatics, RWTH Aachen University, Aachen, Germany; 2 Institute of Neuroscience, Newcastle University, Newcastle Upon Tyne, England, United Kingdom; 3 Faculty of Psychology and Neuroscience, Maastricht University, Maastricht, The Netherlands; McMaster University, Canada

## Abstract

**Background:**

Navigation based on chemosensory information is one of the most important skills in the animal kingdom. Studies on odor localization suggest that humans have lost this ability. However, the experimental approaches used so far were limited to explicit judgements, which might ignore a residual ability for directional smelling on an implicit level without conscious appraisal.

**Methods:**

A novel cueing paradigm was developed in order to determine whether an implicit ability for directional smelling exists. Participants performed a visual two-alternative forced choice task in which the target was preceded either by a side-congruent or a side-incongruent olfactory spatial cue. An explicit odor localization task was implemented in a second experiment.

**Results:**

No effect of cue congruency on mean reaction times could be found. However, a time by condition interaction emerged, with significantly slower responses to congruently compared to incongruently cued targets at the beginning of the experiment. This cueing effect gradually disappeared throughout the course of the experiment. In addition, participants performed at chance level in the explicit odor localization task, thus confirming the results of previous research.

**Conclusion:**

The implicit cueing task suggests the existence of spatial information processing in the olfactory system. Response slowing after a side-congruent olfactory cue is interpreted as a cross-modal attentional interference effect. In addition, habituation might have led to a gradual disappearance of the cueing effect. It is concluded that under immobile conditions with passive monorhinal stimulation, humans are unable to explicitly determine the location of a pure odorant. Implicitly, however, odor localization seems to exert an influence on human behaviour. To our knowledge, these data are the first to show implicit effects of odor localization on overt human behaviour and thus support the hypothesis of residual directional smelling in humans.

## Introduction

Odor localization in humans has repeatedly been subject of recent olfactory research. However, the question whether we are capable, in principle, of creating a spatial representation of the external world purely based on olfactory information is still controversial. Though experience- or gender-based differences in individual performance might exist [1], in laboratory settings people generally only seem to succeed in odor localization if the odorant additionally stimulates the trigeminal nerve [2]. While the olfactory nerve conveys odor-specific chemical information from olfactory sensory neurons, the trigeminal nerve endings are excited by tactile or nociceptive stimuli and relate to the subjective experience of burning or tickling sensations [3]. On the other hand, behavioral experiments have shown that directional smelling can be found in higher vertebrates [4], [5]. In addition, separated sensory epithelia and lateralized neuronal projections into the primary olfactory cortex would in principle allow for spatial comparisons across hemispheres in humans [6], [7].

One reason for the negative findings in human studies might be an inappropriate experimental approach. Former experiments exclusively used explicit judgments to investigate the ability for directional smelling. However, olfaction is known to act to a substantial extent on a preconscious level, which is supposed to be the result of its anatomical and functional organization [8]. Hence, a paradigm that requires the *explicit* assessment of odor location could exceed our capacity for directional smelling. An alternative approach is to assess spatial processing in an implicit way, thereby rendering assessment of conscious access to spatial information unnecessary. A well-known procedure to study implicit spatial processing is the spatial cueing paradigm, which was originally developed by Posner [9], [10] to investigate visual spatial attention. In these paradigms the location of a target is preceded by a cue at either the same (valid cue) or a different location (invalid cue). Typically, people respond faster and more accurately to validly compared to invalidly cued targets. Here, the focus is set on exogenous cueing which relies on the automatic, involuntary spatial orienting response caused by the detection of an otherwise irrelevant cue. These cues do not share any features with the target and do not predict the subsequent target location (for review, see [11]). Since its influential adoption in visuospatial processing, the spatial cueing paradigm has been extended to study the interaction between stimuli of different modalities, including tactile and auditory stimuli. Notably, it appears that a stimulus in one modality can facilitate the processing of a target in a different modality irrespective of task-relevance [12]. For instance, response times and accuracy to tactile stimuli have been shown to be enhanced by visual and auditory cues preceding the target at the same side [13]. Similar findings have been reported for different cue-target relations (e.g. tactile or auditory cues preceding visual targets [13], [14]).

To date, no study has used olfactory stimuli for exogenous spatial cueing of targets in a different modality. Nonetheless, priming procedures have also been used in olfactory research, with many studies demonstrating the high potential of a preceding odorant to enhance processing of subsequent (emotional) stimuli [15]–[23]. However, these priming studies in olfaction have been limited to semantic and emotional contexts, and never included spatial attention.

The present study implemented the spatial cueing paradigm as a novel approach to study lateralization of human olfaction. An olfactory stimulus was used as crossmodal, exogenous cue for a visual target. We hypothesized that a residual ability for directional smelling will become manifest as a spatial cueing effect, with valid cues leading to a significant gain in behavioral performance compared to invalid cues. As women have repeatedly been shown to have better olfactory performance than men [24]–[26], we included sex as a variable of interest in order to investigate a corresponding female advantage in implicit spatial processing of olfactory stimuli. In a second experiment, we sought to replicate former findings of an inability to explicitly localize olfactory stimuli across both nostrils.

## Materials and Methods

### Ethics Statement

The present study was approved by the local ethics committee at the faculty of medicine, RWTH Aachen University, and conducted according to the Code of Ethical Principles for Medical Research involving human subjects of the World Medical Association (Declaration of Helsinki). All subjects gave written informed consent.

### Participants

30 healthy, right-handed volunteers (15 male; mean age 27.0 + 6.7 years) were included in the study. Exclusion criteria leading to impaired olfactory functioning or to structural and/or functional changes in the brain encompassed acute or chronic disorders in the maxillary or frontal sinuses, allergies, smoking, intake of psychoactive substances or medication influencing olfaction, as well as a history of neurological disorders and general psychiatric illness (Structured Clinical Interview for DSM-IV Axis I Disorders SCID-I), or depressive symptoms (Beck Depression Inventory BDI-II [27], a 21-item self-report inventory with a cutoff score of 16). A crystalline intelligence test (WST Wortschatztest [28], a 42-item multiple choice test for the assessment of verbal intelligence and language comprehension) was administered for a rough screening of intelligence. Finally, normal olfactory functioning was ensured by psychophysical testing using the Sniffin' Sticks Screening test (a 12-item odor identification test [29]). Mean scores of the final sample were as follows: 2.3 (+ 2.6) in the BDI, 35.4 (+ 3.0) in the WST, corresponding to a mean IQ of 116.4 (+9.0), and 10.8 (+ 1.0) in the Sniffin' Sticks Screening test, thus ensuring below-cutoff performance in all administered tests.

### Procedure

Participants were seated at a distance of approximately 75 cm in front of the computer screen (14.9″). Visual stimuli were presented via Presentation software (Neurobehavioral Systems, Albany, US). Subjects gave “left” and “right” responses with the index and middle finger of the right hand using a standard computer mouse. The order in which the experiments were presented was fixed across subjects. Neuropsychological, psychopathological and psychophysical screenings were administered between both experiments.

#### Olfactory Stimulation

Odors were delivered using a computer controlled Burghart OM6 olfactometer (Wedel, Germany), which allows standardized olfactory stimulation in the absence of tactile or thermal cues (airflow ∼7l/min). Nostrils were independently stimulated by two separate tubes ending in nose-pieces inserted into the left and right nostril. Continuous airflow through each tube was held constant at ∼7l/min and heated close to body temperature. For each event type, airflow was directed through a separate olfactometer compartment to ensure maximal correspondence between odor and non-odor events, and to rule out any difference in airflow resulting from valve positioning. In addition, white noise was delivered via headphones to prevent any auditory cueing of the olfactory event. Onset and duration of olfactory events were controlled by Presentation software (Neurobehavioral Systems, Albany, US).

For the olfactory cue, phenyl ethyl alcohol (PEA, rose odor; Sigma Aldrich, Schnelldorf; Germany), which is known to act as a pure odorant in reasonably low concentrations [3], [30], was mixed with distilled water in a ratio of 1∶10, and presented in a continuous air stream at a ratio of 3∶4. Distilled water served as control stimulus (ambient air). Subjects were instructed and extensively trained to breathe evenly through the mouth while avoiding nasal air flow (velopharyngeal closure). All participants confirmed perception of the odorant for each nostril independently in a test trial prior to the experiment.

#### Experiment 1 – Implicit spatial cueing task

**Figure 1:**
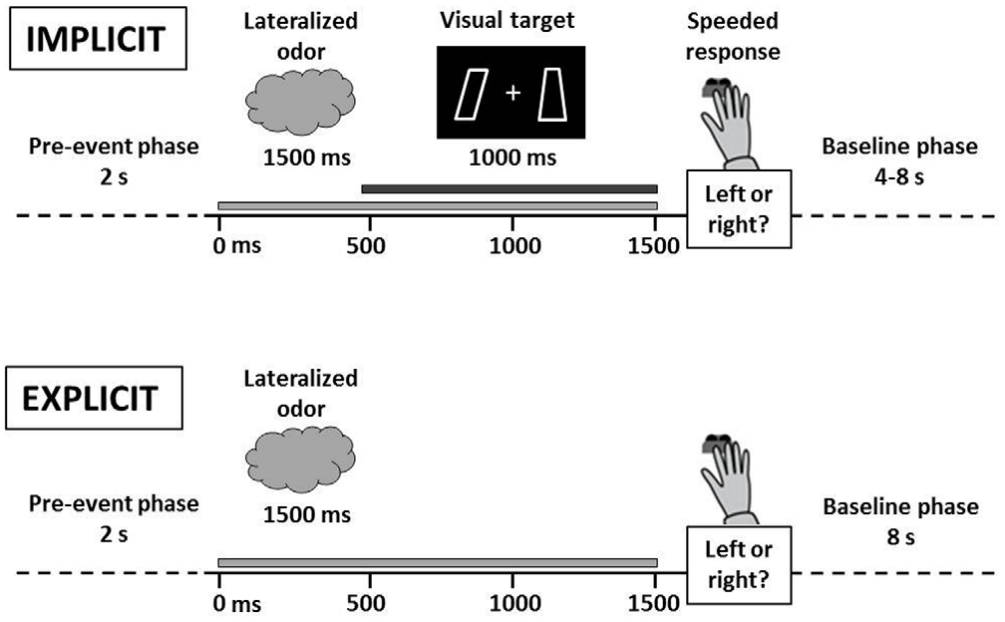
Schematic overview over experimental procedure. In the implicit task, subjects were asked to react as quickly and accurately as possible to a visual target (parallelogram). Prior to target presentation, an olfactory cue was presented either to the same (congruent) or opposite (incongruent) side of the target. In the explicit task, a lateralized odor was presented, and subjects had to indicate the stimulated nostril.

In the olfactory spatial cueing task, participants had to react as quickly and accurately as possible in a side-congruent manner to a visual target stimulus which either appeared to the left or right side of a centrally presented fixation cross (see figure 1, upper part). The order of target lateralization was pseudo-randomized. The target remained visible for 1000 ms. A distracter stimulus was displayed simultaneously at the opposite side to increase task difficulty. Red line drawings of a parallelogram (target) and a trapezoid (distracter) were chosen as visual stimuli, with their centers arranged peripherally at 5.8° visual angle horizontally, and a width and height of 2.0° and 5.9°, respectively. 500 ms prior to visual stimulation, the olfactory cue was presented to the nostril which corresponded either to the side of target presentation (valid cue) or the opposite side (invalid cue) for 1500 ms. Using this stimulation duration, the resulting olfactory mass volume amounted to 6.77 ml, which has been shown to be below trigeminal activation threshold [30]. The odorant cue was non-informative with respect to target location (i.e. exogenous cueing) as it coincided with target position in only 50% of the trials. No olfactory cue was presented during control trials. In total, 90 trials were presented, with 15 trials for each of the six experimental conditions. These conditions resulted from the combination of two factors: 1) side of target (left, right), and 2) olfactory cueing (valid, invalid, control). Each trial consisted of 1) a 2 s pre-event phase, indicated by the color change of the fixation cross, in order to redirect attention to the forthcoming task; 2) an event phase, where the visual target and the preceding olfactory cue were presented for 1500 ms; and 3) a response and baseline phase of 4 to 8 s. Since sensory adaptation is a serious problem in olfactory research, the length of inter-stimulus intervals was chosen in a way that short intervals occurred only in odor-free control trials. Thus, the minimum inter-odor interval (i.e. time elapsed between each successive odor presentation) was 9 s (mean: 12.3 s, maximum: 17.5 s), which has been shown to sufficiently minimize sensory habituation [31].

Prior to the experiment, participants underwent an odor-free training block in order to practice both task and breathing technique and to become accustomed to the olfactometer. In addition, individual reaction times were assessed according to the following rationale: To maximize participants' motivation to respond as quickly as possible in the subsequent spatial cueing experiment, subjects were asked to improve their mean individual reaction time (as assessed in the training block) by 30 ms. Feedback (i.e. happy or sad smiley face) was given after each set of twenty trials. All participants managed to exceed their original response speed, justifying the assumption that they, on average, performed at their maximum speed.

#### Experiment 2 – Explicit localization task

The explicit localization task was implemented as a two-alternative forced-choice procedure in which participants had to decide whether an odorant was presented to the left or to the right nostril (see figure 1, lower part). Both nostrils were stimulated 15 times in a pseudo-randomized order. Trials were designed analogously to Experiment 1. This time, a 1500 ms odor pulse was presented to either the left or right nostril during the event phase. In the subsequent response phase, participants had to indicate their judgment via mouse click. In contrast to the implicit task, the inter-odor-interval was held flexible in the explicit task as it was terminated by the button press. To ensure sufficiently long inter-odor-intervals, minimum trial duration was set to 12.5 s, allowing for a minimum odor interval of 11 s. The maximum trial duration was set to 19.5 s. Thus, the inter-odor-interval varied between 11 and 18 s as a function of individual response time.

### Statistical Analysis

#### Experiment 1 – Implicit spatial cueing task

**Figure 2:**
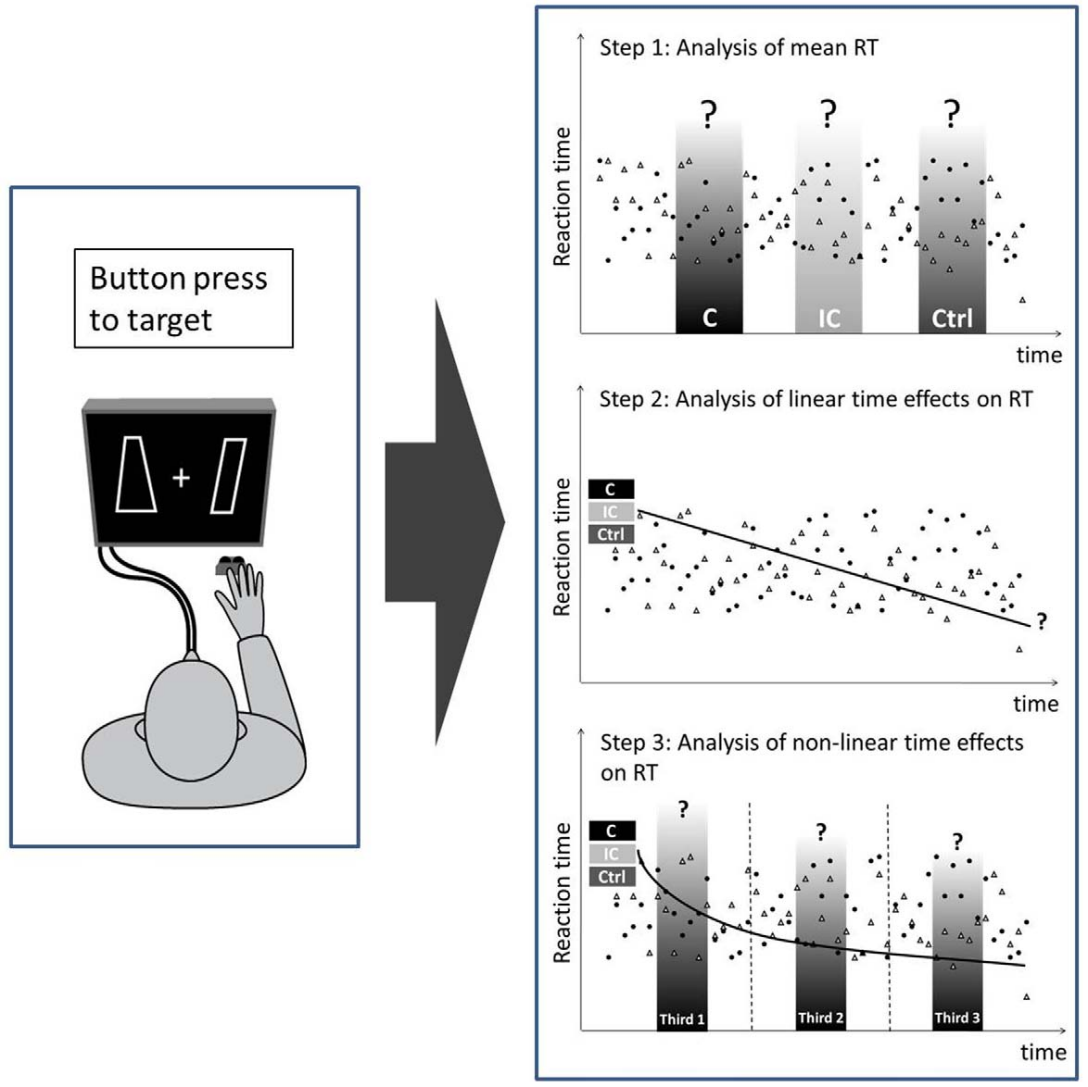
Analysis outline of time effects in the implicit task. In a first step, mean reaction time (RT) to the visual target was averaged across all time points and compared between congruency conditions (congruent, incongruent, control) by means of an rmANOVA. In the second and third step, RT was analyzed as a function of time. A regression approach was used to analyze linear time effects (step 2). Non-linear time effects were explored by comparing mean RT during the first, second and last third of the experiment (step 3).

Dependent variables (DV) were reaction time (RT, in ms) and accuracy (i.e. percentage of correct responses). In order to determine the main effects of experimental manipulation, accuracy and median RTs were subjected to a 3×2×2-way repeated measures analysis of variance (rmANOVA) with within-subject factors “target side” (left vs. right) and “cue” (congruent vs. incongruent vs. control; see figure 2, step 1), and between-subject factor “sex”.

In order to further explore the spatial cueing effect as a function of time, the change in RT throughout the experiment was analyzed with respect to cue congruency. Individual regression analyses with trial number as predictor and RT as dependent variable were computed for the three cueing conditions (congruent, incongruent, control; all 30 trials per condition; see figure 2, step 2). Here, a robust regression procedure was used, as implemented in the MATLAB® command *robustfit*, to reduce the potential influence of outliers on estimates of individual regression coefficients. Statistical analysis was then performed on these slopes by means of a 3×2-way rmANOVA with within-subject factor “cue” (congruent vs. incongruent vs. control) and between-subject factor “sex”. A residual-based outlier removal was introduced, with outliers defined as subjects showing a condition-wise estimated residual which surpasses 2.5 times the standard deviation of the global residual distribution. In addition, planned comparisons were used in order to contrast the effect of one condition against the combined effect of the remaining conditions. The following contrasts were of particular interest: (congruent & incongruent) vs. control (indicated as C&IC > Cont) for assessing the unspecific effect of olfactory stimulation, and congruent vs. incongruent (indicated as C>IC) for assessing the cue-specific effect on target detection. Finally, if applicable, post-hoc analyses were implemented as matched, two-sample t-test comparisons. Adjusted Bonferroni correction for multiple comparisons was applied for the number of calculated tests (i.e.α_crit_ resulting from division of α = .05 by the number of independent tests). In the case that all k factor levels were subjected to post-hoc tests, given a significant result in the overall F-test, the number j of statistically independent tests is k-1 [32]. Except for the robust regression approach, all statistical calculations were performed with SPSS 17.0 (SPSS Inc., Chicago, IL).

#### Experiment 2 – Explicit localization task

Analogously to the implicit task, statistical analyses of explicit localization judgments were conducted on reaction time (RT, in ms) and accuracy (i.e. percentage of correct responses) as DV. Median RT was analyzed by means of a 2×2×2-way rmANOVA with within-subject factors “correctness” (correct vs. incorrect) and “stimulation side” (left vs. right), and between-subject factor “sex”. Likewise, time-dependent effects on RT were explored according to the procedure outlined above: Individual regression slopes on RT were calculated for correct and incorrect trials, respectively. The resulting slopes where then subjected to a 2×2-way rmANOVA with within-subject factor “correctness” and between-subject factor “sex”, after residual-based outlier removal.

Accuracy data were analysed using two different approaches. A 2×2-way rmANOVA with within-subject factor “stimulation side” and between-subject factor “sex” was applied in order to assess average effects of experimental manipulation. In addition, accuracy was analysed within the framework of Signal Detection Theory (SDT) for two-alternative forced-choice paradigms [33]. According to this theory, each trial requires the differentiation between signal (i.e. lateralized olfactory stimulation) and noise (i.e. neutral air delivered in the opposite nostril). For each subject, the following variables were determined: hit rate *h* (i.e. ratio of hits to signal trials), false-alarm rate *f* (i.e. ratio of false alarms to noise trials), sensitivity *d*', and criterion c. Given the two-alternative forced choice scenario, hit rate *h* and false alarm rate *f* are referenced to one of both alternatives (e.g. *h* as hit rate for choosing “right” when “right”, and *f* as false alarm rate for choosing “right” when “left”). The parameter *d*' indicates the distance between both noise and signal distributions, thus approximating the sensitivity of a person. Conversely, the criterion c represents the tendency to favor one side over the other. Parameter estimations for *d*' and c were based on the equal-variance Gaussian model, allowing their values to be calculated from *h* and *f*.

## Results

### Experiment 1 – Implicit spatial cueing task

#### Accuracy

When giving speeded responses to the visual target which was partly preceded by an olfactory cue, subjects performed at a mean accuracy of 96.2% (SD  = 3.0). Accuracy was significantly affected by olfactory cueing (F = 3.748, p = .030), but not by target side (F = 0.450, p = .508) or sex (F = 1.042, p = .316). Planned comparisons revealed an unspecific effect of odor (C&lC > Cont, F = 5.894, p = .022), with higher accuracy in trials preceded by an olfactory cue, but no effect of cue congruency (C>IC, F = 0.213, p = .648; see table 1, figure 2).

**Table 1:** Behavioral performance (mean + standard deviation) in the implicit cueing task and explicit localization task.

implicit	Target side		Cue			Sex	
	Target left	Target right	Congruent	Incongruent	Control	Male	Female
Mean RT	417 + 48	402 + 47	412 + 47	408 + 45	408 + 52	390 + 32	429 + 45
	**F = 8.958,** **p<.006**		F = .890, p<.416			**F = 6.965,** **p<.013**	
Mean accuracy	96.0 + 3.7	96.4 + 4,3	96,5 + 3,1	97.0 + 3.4	95.1 + 5.1	96.4 + 3.0	95.3 + 3.5
	F = .450, p<.508		**F = 3.748,** **p<.030**			F = 1.042, p<.316	

explicit	Stimulation side		Correctness				Sex	
	Left nostril	Right nostril	Correct		Incorrect		Male	Female
Mean RT	1127 + 791	1087 + 796	1073 + 788		1123 + 777		1127 + 727	1049 + 856
	F = .875, p<.357		F = .831, p<.370				F = .001, p<.999	
Mean accuracy	45.8 + 16.9	50.7 + 12.9					47.3 +7.8	49.1 +12.6
	F = 1.666, p<.207						F = .198, p<.660	

Mean RT (in ms) was calculated as arithmetic mean of individual median values. Mean accuracy (% correct) was calculated as arithmetic mean of individual accuracy values. Statistics describe main effects (F value, p value) as derived from rmANOVA, with significant main effects displayed in bold.

#### Reaction time

Average reaction time across all subjects and conditions in the implicit spatial cueing task was 410 (+ 48) ms. Analysis of variance revealed a main effect for target side (F = 8.958, p = .006), with participants responding faster to a right-sided as compared to a left-sided target (see figure 2). A second main effect was found for sex (F = 6.965, p = .013), with men responding faster than women. Crucially, no main effect for cue could be demonstrated (F = 0.890, p = .416), implying that a preceding olfactory cue was, on average, not able to bias target detection (see table 1).

**Figure 3:**
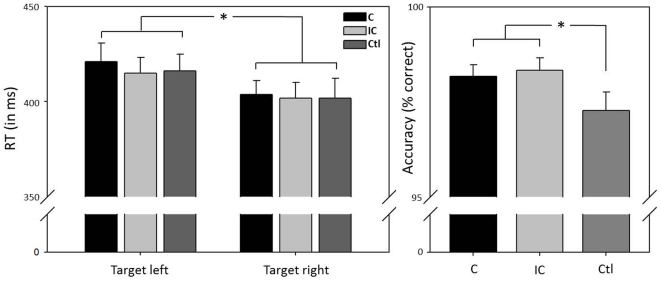
Effects of target side and cue congruency on behavioral performance. Subjects' response (mean RT + SE, in ms) was significantly faster to visual targets presented on the right as compared to targets presented on the left side of the screen. Olfactory cue congruency had no impact on response speed. In contrast, response accuracy (mean accuracy + SE, in % correct) was significantly influenced by olfactory cue congruency, with more accurate responses to cued as compared to non-cued targets. C: congruent, IC: incongruent, Ctl: control, * p<.05.

**Figure 4:**
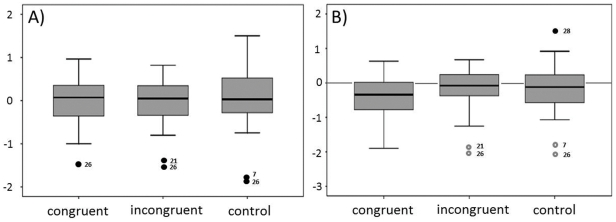
Definition of outliers. For each subject and cueing condition (i.e. congruent, incongruent, control), slopes were calculated using robust regression analysis. Slopes were subjected to a 3×2-way rmANOVA with within-factor “cue” and between-factor “sex”, and the resulting residual distribution was examined. Panel (A) shows condition-wise boxplots of the residuals. Cases deviating 2.5 times the standard deviation from the mean of the global residual distribution (# 7, 21, 26) were defined as outliers. Condition-wise boxplots over the actual slopes confirmed this outlier definition as accurate but less conservative (B, outliers shown in grey).

**Figure 5:**
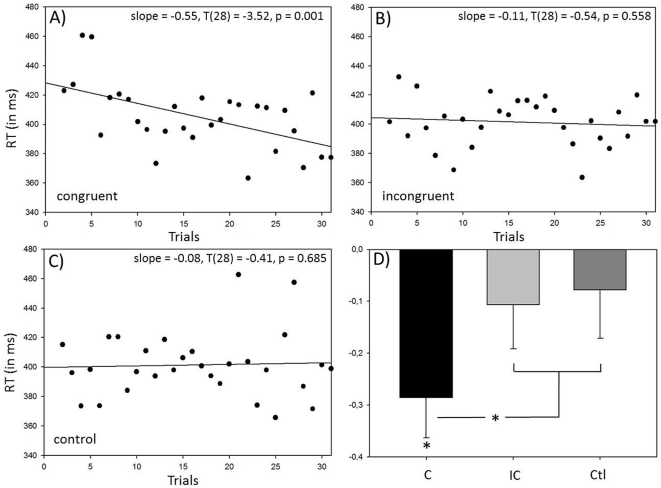
Regression-based analysis of the time-dependent cueing effect. A–C) For illustration of the time-dependent cueing effect, median RT across all subjects was calculated in a trial-by-trial manner, and simple regression slopes were fitted for each condition separately (with standardized slope β and the corresponding statistics of H_0_: β = 0 indicated in the upper right corner of each subplot). The slope in the congruent condition (A) reveals a significant acceleration of RT, based on slower responses at the beginning of the cueing experiment, which is not the case for the incongruent (B) and control (C) condition. D) Mean slope (+ SE) of regression coefficients of the different conditions. A significant difference (* p<.05) was found between the effect of the congruent and the combined effect of the incongruent and control condition (planned comparisons analysis). A significant deviation (* p<.05) from ß = 0 was only found for the congruent condition (one-sample t-test). C: congruent, IC: incongruent, Ctl: control.

However, when time was taken into account by using a linear regression approach, a different picture emerged. Individual regression slopes were calculated separately for each cueing condition. After residual-based outlier removal, which led to the exclusion of three subjects with outlier slopes (see figure 3 A), the rmANOVA revealed a significant effect of cue congruency (F = 3.296, p = .045), thus implying a differential effect of the olfactory cue on the visually-guided RT. Inspection of mean slopes suggests a steeper, negative slope, i.e. RT acceleration, for congruently (beta_congruent_ = −.29 + .40) compared to incongruently (beta_incongruent_ = −.11 + .43) or non-cued control trials (beta_control_ = −.08 + .47; see figure 4 D). Planned comparisons, however, did not show a significant effect of cue congruency (C>IC, F = 3.144, p = .088), or an unspecific effect of odor (C&IC > Cont, F = 3.415, p = .076). Post-hoc analyses between all possible pairs of conditions (C vs. IC, C vs. Cont, IC vs. Cont; j = 2; α_crit_ = .025) revealed a trend for a significant difference between the congruent and control condition (T = −2.179, p = .039), and a less pronounced difference between the congruent and incongruent condition (T = −1.923, p = .066). This was not the case when comparing the incongruent and control condition (T = −.502; p = .620). Subsequent, exploratory one-sample T-tests against a hypothesis of no change over time (beta = 0) suggest that only slopes for validly cued trials were different from 0 (T_congruent_ = −3.815, p<.001). This effect was not observed in the incongruent (T_incongruent_ = −1.196; p = .243), or in the non-cued control condition (T_control_ = −.599; p = .555). For illustrative purposes, median RT data were calculated across subjects in a trial-by-trial manner for the three congruency conditions, and regression slopes were calculated using a simple regression approach. Plots and the corresponding statistics are depicted in figure 5.

**Figure 6:**
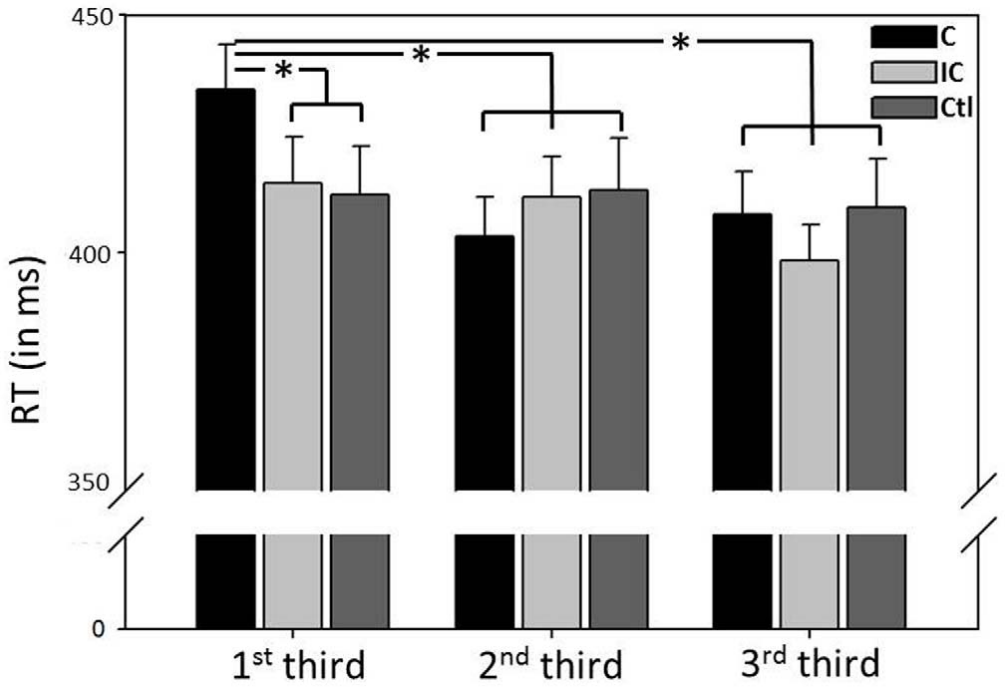
Exploratory analysis of the time-dependent cueing effect. Mean RT (+ SE, in ms) was compared for each cueing condition (congruent, incongruent, control) for the 1^st^, 2^nd^, and last third of the experimental time course. This analysis reveals that subjects initially responded significantly slower to visual targets that were preceded by a side-congruent olfactory cue. This difference disappeared towards the end of the experiment. C: congruent, IC: incongruent, Ctl: control, * p<.05.

In a next step, additional exploratory analyses were conducted in order to further characterize the time-dependent cueing effect. The regression based approach is most sensitive to linear changes of RT, which might ignore non-linear effects on time-courses. . However, Figure 5 implies slower RT for congruently cued trials, which seems to be limited to the beginning of the cueing experiment, thereby resulting in RT acceleration. In order to test for non-linear effects, the time course of the experiment was split into three equivalent parts (see figure 2, step 3). Median RT data was subjected to a 3×3-way rmANOVA, with “thirds” (1st vs. 2nd vs. 3rd third) and “cue” (congruent vs. incongruent vs. control) as within-subject factors. The F-test revealed a significant interaction effect (F = 4.526, p = .002), besides a significant main effect of thirds (F = 3.850, p = .027). To further explore the interaction effect, post-hoc analyses focused on both between-condition effects during the first third (i.e. C_1st_ vs. IC_1st_, C_1st_ vs. Cont_1st_, IC_1st_ vs. Cont_1st_), and within-condition effects for congruently cued trials across thirds (i.e. C_1st_ vs. C_2nd_, C_1st_ vs. C_3rd_, C_2nd_ vs. C_3srd_; j = 6; α_crit_ = .008). A significant between-condition effect was confirmed, as subjects were slower for congruently as compared to incongruently (T = 3.521, p = .001) or non-cued targets (T = 3.350, p = .002) during the first third. No difference was observed when comparing the first third between the incongruent and control condition (T = 0.342, p = .735). In addition, a significant within-condition effect was found for the congruent condition, as RT of the first third of the experiment was significantly slower than RT of the second (T = 4.272, p<.001) and last third (T = 4.017, p<.001). No difference was found between the second and last third (T = −0.681, p = .501; see figure 6).

### Experiment 2 – Explicit localization task

#### Accuracy

When subjects were required to explicitly indicate the side of olfactory stimulation, they performed at a mean accuracy of 48.4% + 10.2. Neither “side of stimulation” (F = 1.666, p = .207) nor “sex” (F = 0.198, p = .660) had significant impact on accuracy, implying that participants, no matter if male or female, failed to perform significantly different from chance when identifying the stimulated nostril (see table 1). The interaction term of “side” and “sex” indicated a tendency for a better localization of right-sided odorants in females (F = 3.404, p = .076). Analysis of accuracy according to SDT revealed a mean sensitivity d' of −0.11 (+ 0.47), and a one-sample t-test showed that the sensitivity to the signal did not differ significantly from zero (T = 0.830, p = .42), suggesting that participants were rather oblivious to the signal. These results also imply the lack of any trigeminal activation for the stimulus delivery (i.e. associated air puffs) and the stimulus itself, thus confirming purely olfactory stimulation. The mean value of the response criterion c was 0.08 + 0.05. A positive value for c points to a tendency to choose right over left, and more subjects fell into the rightward bias category compared to ideal and leftward biased responders. However, this trend was not significant either (T = 1.06, p = .15, one-sided).

#### Reaction time

Mean RT in the explicit odor localization task was 1137 + 803 ms, which is much slower compared to the implicit task as subjects were not required to give a speeded response. No significant main effect of response correctness (F = 0.831, p = .370), stimulation side (F = .875, p = .357), or sex (F = 0.001, p = .999) on RT could be found (see table 1). However, a 3-way interaction emerged (F = 5.709, p = .024), with faster responses to correctly identified left-sided olfactory stimuli in men as opposed to right-sided olfactory stimuli in women. When analyzing time-dependent effects on RT (i.e. acceleration or slowing), regression slopes were calculated for correctly and incorrectly identified trials, respectively. Residual-based outlier removal led to the exclusion of two subjects with outlier slopes. Subsequent analyses of variance revealed no main effect of correctness on RT slopes (F = 0.680, p = .417). However, a main effect of sex emerged (F = 4.902, p = .036), with post-hoc tests revealing a slower response at the beginning of the experiment, and hence a stronger acceleration for men (slope_male_ = −11.07 + 12.30) than for women (slope_female_ = −1.21 + 13.68; T = 2.004, p = .056).

## Discussion

### Summary of main results

In this study, a novel approach using olfactory spatial cues was successfully implemented to investigate implicit processing of olfactory spatial information. The expected effect of an altered reaction to congruently or incongruently cued targets was not found when analysing mean RT. However, a significant time by condition interaction indicates that the initial response to a visual target was markedly altered when an olfactory cue was presented at the same side. This effect resulted exclusively from the spatial congruency of a left- or right-sided olfactory cue, which points to a residual ability for directional smelling in humans. In contrast, explicit judgment of laterality resulted in chance performance, which replicates former findings.

### Discussion of the olfactory cueing effect

In general, the olfactory cue seems to induce an unspecific alerting effect independent from location, as accuracy for cued targets was significantly higher than for non-cued targets. In addition, our finding of a slowed response to congruently cued targets was somewhat unexpected, as congruent cues usually lead to response facilitation [9], [10]. As such, it seems that the notion of cue validity or (spatial) congruency has to be revised for the olfactory modality. Neuroanatomically, the olfactory system differs from other sensory systems with respect to the lateralization of afferent fibres. The olfactory tract projects mainly ipsilaterally into the olfactory cortex without thalamic intermediary [7], which differs from the wiring pattern of other modalities, and might be the reason for the adverse spatial cueing effect. What does spatial congruency thus mean with respect to the olfactory modality? The reported data suggest that an olfactory cue presented at the same side as a visual target leads to interference. As this effect is solely based on the location, and not on the mere presence of the cue, an interaction of visual and olfactory spatial processing must have occurred.

Different kinds of cue-target interactions have been reported in the literature. According to the ‘hemispheric-activation’ account [34], [35], the processing of the olfactory cue might result in a spread of activation within the ipsilateral hemisphere, and consequently to a shift of spatial attention to the corresponding, i.e. contralateral, visual hemifield. The congruent target would thus appear in the unattended hemifield, resulting in slower processing. However, studies on cross-modal spatial attention demonstrated that cross-modal links in spatial attention are better accounted for by a common, external reference space [36], [37]. As such, the most prominent cue-target interaction is the automatic induction of a spatial bias by the cue, which leads to enhanced detection of a congruent target [9], [10]. If this was applicable to the olfactory-visual scenario, the olfactory cue would have created a spatial bias which directed the subject's attention *away* from the stimulated side, as responses were slower in the congruent condition. Although this appears highly counterintuitive, a recent study reported a significant bias towards the not stimulated nostril when localizing unilaterally administered vanillin, which is known to act as a pure olfactory stimulus [30]. Although the authors speculated this to be a chance phenomenon, it is interesting in the light of the present cueing effect. Another well-known effect is the so-called inhibition of return (IOR), which refers to longer reaction times to targets following a side-congruent cue after a delay of 300 ms or more [38]. This is interpreted as an attentional shift away from the cued location where no target appeared during the critical period [39], and has also been shown in cross-modal spatial cueing paradigms. As the cue-target interval in our paradigm was 500 ms, it can be speculated that this supra-modal process applies to the olfactory modality as well. Future studies are encouraged to further explore the contribution of supra-modal and modality-specific constraints to cross-modal spatial attention involving the olfactory domain.

Another finding which contradicts traditional cueing results are the temporal characteristics of the reported olfactory cueing effect. Response slowing to congruently cued targets occurred only during the first third of the experiment. This indicates that the assumed interaction of visual and olfactory spatial information processing gradually disappears. A possible time-dependent effect underlying this disappearance is learning. In other words, subjects might have learned to ignore the interfering cue. However, as learning is supra-modal, this fading cueing effect would be expected in paradigms involving other modalities as well, which, to our knowledge, has not been reported before. In addition, Wysocki [40] has ruled out learning effects for odor localization in particular, as localization accuracy did not improve after training with and without feedback. An alternative time-dependent process is habituation [41], which is a particularly pronounced in the olfactory modality. After repeated or prolonged exposure to odors, the neural sensitivity towards a stimulus is diminished, rendering the stimulus low in salience and, consequently, low in cueing power [42]. Although the odor cue in the present study consisted of a short pulse of 1s, the inter-stimulus interval (ISI) was chosen to be relatively short, ranging from 9 to 17.5 s. It is likely that habituation occurred during this olfactory cueing task, which is thus a candidate process for causing a gradual disappearance of the spatial cueing effect.

### Olfactory localization: background and former findings

Albeit the exact mechanism of the observed cueing effect remains a matter of debate, the central finding of this study argues for the existence of spatial processing in the olfactory modality. In previous experiments, investigation of the effects of odor localization was limited to explicit ratings as in experiment 2. The findings implied that the human olfactory system has lost the ability of directional smelling, and that activation of the trigeminal nerve is crucial for localizing odorants in space [1], [2], [43], [44]. However, a number of observations argue that our phylogenetic heritage has not entirely disappeared. Using behavioural measurements and functional magnetic resonance imaging (fMRI), Porter and colleagues [45] reported a localization accuracy of >70% for PEA, which was associated with activation in the right piriform cortex. Frasnelli et al. [1] also observed successful localization of PEA in a subset of their study sample. The authors raised the question whether the ability to localize odors is a function of individual experience, which in turn could be improved by training and behavioural demands. Relating to this, another study could demonstrate the impact of experimental settings on odor localization [43]. In a passive condition, unilaterally presented olfactory stimuli were delivered to the subjects nostrils, whereas in the active, more naturalistic condition, subjects had to actively sniff the odors. Although pure olfactory stimuli were not localizable above chance level in either condition, an improvement of localization accuracy by active sniffing was found for pure odorants, but not for mixed olfactory/trigeminal stimuli. All these findings point to a possible residual ability for directional smelling in humans, which might be difficult to assess using common approaches. As such, the current data argue for implicit testing as a suitable means, which is justified by two reasons. First, the sense of smell is often regarded as “hidden sense” as it primarily acts beyond consciousness [46], presumably due to its unique anatomical (i.e. no thalamic intermediary; [8], [47]) and physiological (i.e. rapid central and peripheral sensory adaptation; [48], [49]) properties. Second, the absence of an effect on overt behaviour does not preclude the existence of an effect on a preconscious level [50]. For directional smelling, residual abilities seem to be too weak to exert their influence explicitly under untrained, artificial conditions. However, they might have significant effects on a pre-attentive level, as suggested by our spatial cueing results.

### Discussion of other effects

A robust effect observed in the implicit spatial cueing experiment was a significant difference of RT across sides of visual target presentation, with “right” responses being on average given more quickly than “left” responses. This likely represents a side effect resulting from lateralization processes, as all responses were given with the right hand (Poffenberger effect; Poffenberger, 1912; see [51] for review). More interesting with regard to the olfactory modality is the impact of gender differences. Some studies have reported superior olfactory performance in women in basic sensory processing [52]–[54], as well as higher order olfactory tasks [24]. In contrast, no differences between men and women have been demonstrated for odor localization so far [1], [43], [55]–[57]. which holds true for the current study. The main effect of sex in both experiments was merely related to the motor response. Men responded faster in the implicit cueing task, but were initially slower in the explicit localization task. Possibly, men were more indecisive about which button to press, given the uncertainty of the olfactory stimulation. Even more difficult to interpret is the finding in the explicit localization task that women showed a significantly faster response to correctly identified stimuli presented on the right side, which was exactly reversed in men. This effect might represent a chance finding, but might also result from lateralization differences between sexes. Interestingly, two electrophysiological investigations about the influence of sex on olfactory lateralization could show reversed processing patterns between men and women, both with unilateral and bilateral stimulation [58]–[59]. Although these studies did not investigate speeded responses to olfactory stimulation, their findings justify speculations about corresponding differences on a behavioural level.

### Limitations

The central limitation of the current study was the choice of a rather short inter-odor interval, which very likely resulted in habituation. However, experiments involving speeded responses in order to assess the effect of interest require the subjects to perform at maximum speed, which in turn sets limits to the overall duration of the experiment. As such, another study with speeded responses to odors used a similarly short inter-odor interval, and also referred to habituation as a possible limitation [60]. Future studies should focus on how to solve this dilemma of minimizing habituation while maximizing the subject's alertness and, in turn, response speed. Another limitation is the unnatural setting of the experiment. It would be interesting to investigate whether active sniffing, and the presentation of a less steep odor gradient (i.e. not 100% exposure to one nostril only but rather a strong odor pulse to one nostril and a minor pulse to the other one), would lead to a more pronounced effect of the olfactory cue. By presenting the odor to only one nostril, the other nostril is prevented to contribute to the relevant neural computations, and thus may be functionally blindfolded, much like the blocking of one ear leads to deterioration of source localization in auditory task [61]. Finally, the inclusion of a trigeminal-visual cueing paradigm would have been highly interesting, as a robust cueing effect can be hypothesized to occur with trigeminal cues. This could serve as a control condition to investigate to what extent spatial cueing can be adapted to the chemosensory modality in general.

### Conclusion

The results of a novel approach to study olfactory spatial representations point to the existence of residual directional smelling abilities in humans. Using implicit testing, spatial processing of pure odorants exerted an influence on behavioural performance, which did not become manifest when assessing explicit judgments about odor location. These findings imply an increased sensitivity of implicit tests for spatial abilities in human olfaction. Given a possible modulation of this ability by experience (e.g. training), this approach may help to reconcile conflicting findings about directional smelling in previous studies. Our results also extend the existing body of research on crossmodal cueing, which up to now did not include the chemosensory modality. Further studies are encouraged to determine in more detail the commonalities and differences of spatial cueing effects using chemosensory stimuli compared to stimuli of other modalities.
